# Fibulin-3 as a biomarker of response to treatment in malignant mesothelioma

**DOI:** 10.1515/raon-2015-0019

**Published:** 2015-08-21

**Authors:** Viljem Kovac, Metoda Dodic-Fikfak, Niko Arneric, Vita Dolzan, Alenka Franko

**Affiliations:** 1 Institute of Oncology Ljubljana, Zaloška cesta 2, Ljubljana, Slovenia; 2 Clinical Institute of Occupational Medicine, University Medical Center Ljubljana, Ljubljana, Slovenia; 3 Pharmacogenetics Laboratory, Institute of Biochemistry, Faculty of Medicine, University of Ljubljana, Ljubljana, Slovenia

**Keywords:** fibulin-3, biomarker, malignant mesothelioma, response to treatment

## Abstract

**Background:**

Fibulin-3 is a new potential biomarker for malignant mesothelioma (MM). This study evaluated the potential applicability of fibulin-3 plasma levels as a biomarker of response to treatment and its prognostic value for progressive disease within 18 months. The potential applicability of fibulin-3 in comparison with or in addition to soluble mesothelin-related peptides (SMRP) was also assessed.

**Patients and methods.:**

The study included 78 MM patients treated at the Institute of Oncology Ljubljana between 2007 and 2011. Fibulin-3 levels in plasma samples obtained before treatment and in various responses to treatment were measured with the enzyme-linked immunosorbent assay.

**Results:**

In patients evaluated before the treatment, fibulin-3 levels were not influenced by histopathological sub-types, tumour stages or the presence of metastatic disease. Significantly higher fibulin-3 levels were found in progressive disease as compared to the levels before treatment (Mann-Whitney [U] test = 472.50, p = 0.003), in complete response to treatment (U = 42.00, p = 0.010), and in stable disease (U = 542.00, p = 0.001). Patients with fibulin-3 levels exceeding 34.25 ng/ml before treatment had more than four times higher probability for developing progressive disease within 18 months (odds ratio [OR] = 4.35, 95% confidence interval [CI] 1.56–12.13). Additionally, patients with fibulin-3 levels above 34.25 ng/ml after treatment with complete response or stable disease had increased odds for progressive disease within 18 months (OR = 6.94, 95% CI 0.99–48.55 and OR = 4.39, 95% CI 1.63–11.81, respectively).

**Conclusions:**

Our findings suggest that in addition to SMRP fibulin-3 could also be helpful in detecting the progression of MM.

## Introduction

Malignant mesothelioma (MM) is an aggressive malignant disease that has been associated with occupational and environmental exposure to asbestos.^1^–^8^ Most commonly it arises from serosal cells of the pleura and less frequently from peritoneum or other serosal surfaces such as pericardium and tunica vaginalis.^6^,^9^

Malignant mesothelioma remains a fatal disease that is hard to treat with favourable outcome.^9^,^10^ Hence, potential new biomarkers for earlier diagnosis and following the response to treatment have been intensively investigated. One of the most extensively studied blood-based biomarkers is soluble mesothelin-related peptides (SMRP); however, the poor sensitivity limits its added value to early diagnosis.^10^,^11^ Nevertheless, the results of our previous study suggest that SMRP may be a useful tumour marker for detecting the progression of MM and evaluating tumour response to treatment.^12^

Fibulin-3, also known as epidermal growth factor containing fibulin-like extracellular matrix protein 1 (EFEMP1), is suggested to be a new potential biomarker for MM.^13^ Fibulin- 3 belongs to a family of extracellular matrix glycoproteins^14^ that have recently been shown to act as tumour suppressors or activators in different cancers.^15^–^17^ It has restricted expression in the body and is predominately localized in the extracellular matrix of elastic tissue.^18^ The levels of fibulin-3 expression have been found to be decreased in many cancer types due to promoter hypermethylation and have been correlated with poor survival of patients with lung cancer^19^,^20^, breast cancer^21^, and hepatocellular carcinoma.^22^ On the other hand, an increase in fibulin-3 was observed in malignant gliomas^23^, cervical carcinomas^24^, and pancreatic cancer.^25^

Fibulin-3 was first studied as a biomarker of MM by Pass *et al*. who reported that plasma fibulin-3 levels can distinguish a healthy person with exposure to asbestos from patients with MM.^13^ They found that in conjunction with fibulin-3 levels in pleural effusions, plasma fibulin-3 levels can further differentiate MM effusion from other malignant and benign effusions.^13^ Recent studies identified soluble mesothelin as a superior diagnostic biomarker for MM compared to fibulin-3, whereas fibulin-3 provided superior prognostic information compared to mesothelin.^26^

According to our knowledge and available literature, fibulin-3 has not been studied so far as a biomarker for evaluating tumour response to treatment. This study aimed to determine fibulin-3 levels in plasma of patients with MM before treatment and in various responses to treatment (complete response, partial response, stable disease, and progressive disease), to evaluate its potential applicability as a biomarker of tumour response to treatment, and to assess if plasma level of fibulin-3 could predict the probability of progressive disease after the response to treatment in the period of 18 months. We also assessed the potential applicability of fibulin-3 as a biomarker of tumour response to treatment in comparison with or in addition to SMRP.

## Patients and methods

### Patients

A panel study was performed. Patients eligible for inclusion in the study had histologically proven MM and each subject acted as her/his own control in an ongoing longitudinal study.^12^ Briefly, the study included 78 patients with MM treated at the Institute of Oncology Ljubljana in the period between March 2007 and June 2011.

Eligibility criteria included biopsy-proven MM. In all patients, thoracoscopy or laparoscopy/laparotomy was performed. The immunohistochemistry methods were used (Cytokeratin 5/6 [CK5/6], Epithelial Membrane Antigen [EMA], Calretinin, Vimentin, Wilms tumour gene–1 [WT1], CD15, Ber-EP4, B72.3, MOC-31, actin, desmin, S-100, Carcinoembryonic Antigen [CEA], thyroid transcriptor factor1 [TTF-1]). The patients had no history of another cancer during the past 5 years or breast cancer ever; the Eastern Cooperative Oncology Group (ECOG) performance status (PS) was 0–2.

Tumour extension was classified according to TNM classification, based on the results from chest and upper abdominal CT scan and thoracoscopy.^27^ For comparison with subsequent scanning, the thickness of the tumour on three CT levels was recorded considering the modified RECIST criteria.^28^ Sporadically a NMR was done to evaluate the operability of some patients^29^ and a PET-CT was also done in some patients to evaluate the extent of disease and response to treatment like in patients with lung cancer.^30^

The patients were treated with 4 to 9 cycles of chemotherapy comprising cisplatin and low dose gemcitabine in prolonged infusion, or cisplatin and pemetrexed.^31^–^34^ In one patient with pleural MM, extrapleural pleuropneumonectomy was carried out before chemotherapy and in four patients with pleural MM, it was carried out after chemotherapy. Peritonectomy was performed in two patients with peritoneal MM before chemotherapy and in three patients after chemotherapy. Four patients received best supportive care only. Twenty-nine patients with pleural MM were treated with second-line chemotherapy and two of them received palliative radiotherapy.^12^

For all the patients, data on smoking were obtained using a standardized questionnaire. The duration of smoking and the number of pack-years of smoking were calculated for each subject.^35^,^36^ To determine occupational and/or environmental asbestos exposure, a semi-quantitative method was used as previously described.^12^

## Methods

Blood specimen collection was carried out in patients before treatment (before the 1^st^ cycle of chemotherapy or surgery) and/or after treatment (after the third and/or the sixth cycle of chemotherapy or surgical procedure) and/or at the progress of the disease. In total, 135 blood samples from 78 patients were collected in different periods of disease and treatment.

Plasma was prepared immediately after blood sampling and stored in aliquots frozen at −30 ºC until the fibulin-3 assay was performed. Fibulin-3 levels in plasma were measured with the use of enzyme-linked immunosorbent assay (Uscn Life Science Inc., Wuhan, China). The median value of fibulin-3 in complete response or after the surgery was chosen as the cut-off level.

For all the patients, the information on SMRP levels was available from our previous study^12^ for the same time-points before and/or after treatment. A level of 1.50 nmol/L was considered as a cut-off value for positive SMRP. Using receiver operating characteristic (ROC) curve analysis, we determined the fibulin-3 cut-off values for prediction of disease progression. We compared serum levels in progressive disease with levels in complete response, partial response or stable disease and calculated the area under the curve (AUC), sensitivity and specificity. As our aim was to determine the usefulness of serum fibulin-3 for screening for progressive disease, cut-off value with at least 80% sensitivity was selected to limit the potential for false negative results. On the other hand, lower specificity would not be as problematic, as patients would have a more detailed check-up after initial screening.

### Statistics and ethical consideration

Standard descriptive statistics were used to describe each variable. Mann-Whitney test (U) test was performed to determine the differences in fibulin-3 levels before treatment and in various responses to treatment. The correlations between fibulin-3 and SMRP levels were calculated using Pearson’s correlation coefficient. Logistic regression analysis was used to assess the odds for different responses to treatment.

Prior to inclusion, all patients were fully informed about the study and signed informed consent to participate. The study was approved by the Slovenian Ethics Committee for Research in Medicine and was carried out according to the Helsinki Declaration.

## Results

### Patients

The study included 78 patients with MM, 57 (73%) male and 21 (27%) female. The overall median (min–max range) age was 66 (23–84) years. Among them, 35 (44.9%) were ever smokers and 43 (55.1%) of them never smoked. The median duration of smoking was 18 (1–69) years, the median number of smoked cigarettes per day was 20 (1–29) and the median pack-years of smoking amounted to 15 (1–45).

Asbestos exposure was confirmed in 67 (85.9%) of the patients with MM. The assessed exposure was low in 24 (30.8%) patients, median in 21 (26.9%) patients, and high in 22 (28.2%) patients, while in 11 patients (14.1%) asbestos exposure could not be proven with certainty. In the exposed group, the median duration of exposure was 90.50 (0.1–528) months.

Regarding the location of the disease, 70 (89.7%) patients had pleural and 8 (10.3%) peritoneal MM. Epitheloid MM was found in 64 (82.0%), biphasic in 7 (9.0%), and sarcomatoid in 7 (9.0%) patients. Five (6.4%) patients were diagnosed with stage I, 17 (21.8%) with stage II, 28 (35.9%) with stage III and 20 (25.6%) with stage IV, while 8 (10.3%) patients had MM of peritoneum and therefore, the stage could not been determined. The median survival of all patients was 20.1 (2.8–86.1) months.

Among 33 patients evaluated before treatment, no significant differences in fibulin-3 levels were observed between histopathological subtypes or between tumour stages. Fibulin-3 levels before treatment were not significantly different between patients with and without evidence of metastatic disease (U = 73.00, p = 0.854) (Table 1).

The results of descriptive statistics for fibulin-3 levels before treatment and/or in different responses to treatment for all 78 malignant mesothelioma patients are presented in Table 2. No significant difference was observed between the fibulin-3 levels before treatment as compared to the levels in patients in complete response to treatment (U = 71.00, p = 0.641), partial response to treatment (U = 186.00, p = 0.496), or stable disease (U = 597.00, p = 0.603). On the other hand, significantly higher fibulin-3 levels were found in progressive disease as compared to the levels before treatment (U = 472.50, p = 0.006). Fibulin-3 levels were also significantly higher in progressive disease as compared to the levels in complete response to treatment (U = 42.00, p = 0.020) or stable disease (U = 542.00, p = 0.002), while no significant difference was observed between progressive disease and partial response to treatment (U = 229.00, p = 0.241).

No correlation (r = 0.364, p < 0.001) was detected between fibulin-3 levels and SMRP levels as determined at the same time-points in our previous study.^12^

**FIGURE 1.**
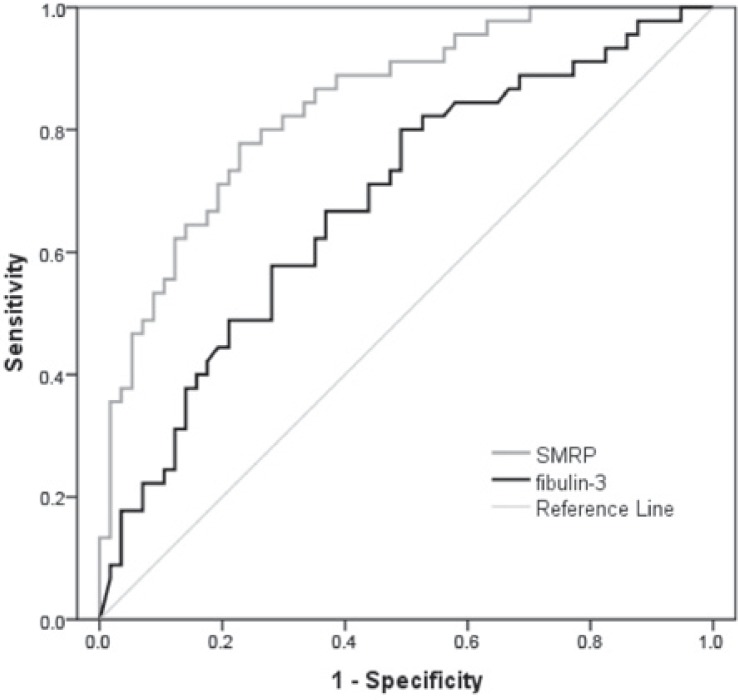
Receiver operating characteristic (ROC) curve for mesothelin-related peptides (SMRP) and fibulin-3 serum levels comparing values at progressive disease with values at complete response, partial response or stable disease.

In ROC curve analysis comparing progressive disease with complete response, partial response or stable disease, AUC for fibulin-3 was 68.3% (95% CI = 57.9–78.7, p = 0.002). Cut-off value of 34.25 ng/ml had sensitivity of 82.2%, thus passing the sensitivity threshold of 80%. On the other hand, specificity for this cut-off value was 47.7% (Figure 1). For mesothelin levels, AUC was 84.2% (95% CI = 76.8–91.7, p < 0.001, Figure 1). Previously determined cut-off value of 1.5 nmol/L had high sensitivity of 91.1% and specificity of 49.1%, thus also limiting the chance of false negative results.

For further logistic regression analysis, fibulin-3 levels were categorized into two categories based on ROC curve analysis: ≤ 34.25 ng/ml and > 34.25 ng/ml. Patients with fibulin-3 levels before treatment exceeding 34.25 ng/ml had more than four times higher probability for developing progressive disease during the period of 18 months (OR = 4.35, 95% CI 1.56–12.13, p = 0.005). However, fibulin-3 levels before treatment were not associated with complete response to treatment (OR = 0.63, 95% CI 0.09–4.26, p = 0.633), partial response to treatment (OR = 1.51, 95% CI 0.41–5.58, p = 0.540), or stable disease (OR = 0.99, 95% CI 0.39–2.51, p = 0.984). Nevertheless, patients with fibulin-3 levels higher than 34.25 ng/ml after the treatment with complete response to treatment or with stable disease showed increased odds for developing progressive disease during the period of 18 months (OR = 6.94, 95% CI 0.99–48.55, p = 0.051 and OR = 4.39, 95% CI 1.63–11.81, p = 0.003 respectively) (Table 3).

The analysis also showed that patients with pre-treatment SMRP levels >1.50 nmol/L had almost six times higher odds for progressive disease during the period of 18 months (OR = 5.86, 95% CI 1.68–22.40, p = 0.005). Additionally, patients with SMRP levels >1.50 nmol/L after the treatment and with complete response to treatment, partial response to treatment, and stable disease were at higher risk for developing progressive disease compared with those with SMRP ≤ 1.50 nmol/L during the period of 18 months (OR = 41.00, 95% CI 3.65–461.03, p = 0.003, OR = 8.79, 95% CI 1.97–39.28, p = 0.004, and OR = 7.92, 95% CI 2.37–26.46, p = 0.001 respectively) (Table 3).

To evaluate the combined effect of fibulin-3 and SMRP levels in evaluating tumour response to treatment, we constructed multivariate logistic regression models that included two categories of fibulin-3 (> 34.25 ng/ml vs. ≤ 34.25 ng/ml) and two categories of SMRP (>1.50 nmol/L vs. ≤ 1.50 nmol/L). The odds for developing different responses to treatment (complete response, partial response, stable disease, progressive disease) did not change considerably compared to the results of univarate logistic regression analysis when pretreatment fibulin-3 and SMRP were both above the respective cut-off levels (34.25 ng/ml and 1.50 nmol/L respectively). Similarly, the probability for developing progressive disease did not change significantly compared with the results of univarate logistic regression analysis when fibulin-3 and SMRP were both above the respective cut-off levels in different responses to treatment (Table 3).

## Discussion

As expected the occupational and/or environmental exposure to asbestos was confirmed in almost 86% of patients with MM. This is in agreement with the results of the studies published so far that have proposed asbestos as a major cause for developing this aggressive disease.^1^–^8^

Fibulin-3 has recently been suggested as a new tumour biomarker for MM.^13^,^26^ Pass *et al*. presented that plasma fibulin-3 levels can distinguish an asbestos exposed healthy person from patients with MM.^13^ Creaney *et al*. recognized soluble mesothelin as a superior diagnostic biomarker for MM compared with fibulin-3, while fibulin-3 was indicated to provide superior prognostic information compared with mesothelin.^26^ However, to our knowledge and available literature, fibulin-3 has not been studied yet as a biomarker for evaluating tumour response to treatment.

The results of the current study show significantly higher fibulin-3 levels in progressive disease as compared with the levels before treatment, in complete response to treatment, and in stable disease, which indicates that fibulin-3 could be helpful in identifying the progression of MM. On the other hand, no significant difference was observed between the fibulin-3 levels before treatment as compared with the levels in complete response to treatment, partial response to treatment, and stable disease. The results of our previous study investigating SMRP as a tumour biomarker for MM, showed significantly higher SMRP levels before treatment than the levels in complete response, partial response, and a borderline significant difference between levels before treatment and stable disease.^12^ These findings suggest SMRP not only as a superior diagnostic biomarker for MM compared with fibulin-3 as presented in the study of Creaney *et al*.^26^, but also as a superior biomarker for evaluating tumour response to treatment.

An important finding of the current study shows that the probability for the development of progressive disease during the period of 18 months was more than four times higher when fibulin-3 levels before treatment exceeded 34.25 ng/ml, and almost five times higher when SMRP level before treatment was higher than 1.50 nmol/L. The analysis also showed increased odds for developing progressive disease during the period of 18 months when fibulin-3 levels after the treatment and with complete response to treatment or stable disease were higher than 34.25 ng/ml. The same holds true of SMRP levels above 1.50 nmol/L in complete response, partial response, and stable disease. These results suggest that in addition to SMRP, the fibulin-3 levels before treatment, in complete response to treatment, and in stable disease could help predict the risk of developing progressive disease in MM. When including fibulin-3 levels above 34.25 ng/ml and SMRP levels above 1.50 nmol/L in multivariate logistic regression models, the odds for both fibulin-3 and SMRP did not change significantly, suggesting independent effects. However, we have to indicate that the confidence intervals were wide because the number of involved subjects was low.

In conclusion, the findings of our current study show that in addition to SMRP^12^, fibulin-3 could also be helpful in detecting the progression of MM. Contrary to SMRP^12^, fibulin-3 has not been proven as a useful biomarker for evaluating tumour response to treatment. The results of the present study also indicate that fibulin-3 levels before treatment, in complete response to treatment, and in stable disease could be beneficial in predicting the risk of developing progressive disease in patients with MM. To increase the power of the study and to validate these results, a larger sample size is needed.

## Figures and Tables

**TABLE 1.** Fibulin-3 levels (ng/ml) before treatment at different histopathological subtypes, at different tumour stages and according to the presence of metastatic disease in patients with malignant mesothelioma

**Characteristics Subtype**	**Mean**	**SD**	**Median**	**Range**	**Inter-quartile**	**Mann-Whitney (U) test**	***p* value**
Epitheloid (N = 25)	41.52	24.26	36.42	1.65–92.32	23.00–58.14	69.00^a^	0.789
Biphasic (N = 6)	41.04	16.57	35.24	22.72–65.22	28.41–59.41	2.00^b^	0.286
Sarcomatoid (N = 2)	27.36	1.60	27.36	26.23–28.49	26.23–27.36	17.50^c^	0.519

**Tumour stage^d^**							
I^e^ (N = 1)							
II (N = 8)	28.88	8.32	28.67	15.69–40.78	21.98–35.32	32.00^f^	0.156
III (N = 13)	46.39	28.09	47.41	1.65–92.32	22.03–66.43	42.00^g^	0.817
IV (N = 7)	40.97	21.71	30.31	22.72–84.33	28.49–54.93	19.00^h^	0.320

**Metastatic disease**							
Present (N = 7)	40.97	21.70	30.31	22.72–84.33	28.49–54.93	73.00^i^	0.854
Not present (N = 22)	39.89	23.38	35.25	1.65–92.32	22.21–54.12		

N = number of plasma samples;

a Mann-Whitney (U) test calculated for epitheloid subtype vs. biphasic subtype;

b Mann-Whitney (U) test calculated for biphasic vs. sarcomatoid subtype;

c Mann-Whitney (U) test calculated for epitheloid subtype vs. sarcomatoid subtype;

d Pleural malignant mesothelioma only;

e Stage I was found only in one patient with fibulin-3 level 43.44 ng/ml;

f Mann-Whitney (U) test calculated for stage II vs. III;

g Mann-Whitney (U) test calculated for stage III vs. IV;

h Mann-Whitney (U) test calculated for stage II vs. IV;

i Mann-Whitney (U) test calculated for metastatic disease present vs. not present

**TABLE 2.** Fibulin-3 levels (ng/ml) before treatment and in different responses to treatment in 78 patients with MM

**Disease phase**	**Mean**	**SD**	**Median**	**Range**	**Inter-quartile**	**Mann-Whitney (U) test**	***p* value**
**All phases** (N = 135)	44.57	21.31	40.78	0.00–105.00	29.18–56.27		
**Before treatment** (N = 33)	40.57	22.26	35.09	1.65–92.32	24.23–56.21	877.00^a^	0.598
**Complete response or after surgery (N = 5)**	32.43	9.98	34.25	18.16–45.50	23.55–40.40		
**Partial response** (N = 13)	45.13	26.48	41.18	0.00–105.00	27.90–56.42		
**Stable disease** (N = 39)	40.00	16.11	37.10	6.52–73.44	29.40–47.56		
**Progressive disease** (N = 45)	53.56	21.67	47.19	16.26–105.00	37.78–67.93	813.00^b^	0.001

N = number of plasma samples;

a Mann-Whitney (U) test calculated for fibulin-3 before treatment vs. stable disease + partial response + complete response or after surgery;

b Mann-Whitney (U) test calculated for fibulin-3 in progressive disease vs. stable disease + partial response + complete response or after surgery

**TABLE 3.** The odds for developing different responses to treatment for fibulin-3 levels > 34.25 ng/ and SMRP levels >1.50 nmol/L in univariate and multivariate analysis

	**Univariate analysis**		**Multivariate analysis**	

	**Fibulin-3 OR (95% CI)**	**SMRP OR (95% CI)**	**Fibulin-3 OR (95% CI)**	**SMRP OR (95% CI)**
**Before treatment vs. complete response**	0.63 (0.09–4.26)	0.14 (0.01–1.43)	0.74 (0.1–5.48)	0.15 (0.02–1.48)
**Before treatment vs. partial response**	1.51 (0.41–5.58)	0.67 (0.18–2.45)	1.56 (0.42–5.84)	0.64 (0.17–2.39)
**Before treatment vs. stable disease**	0.99 (0.39–2.51)	0.74 (0.29–1.91)	1.04 (0.41–2.67)	0.73 (0.28–1.93)
**Before treatment vs. progressive disease**	4.35 (1.56–12.13)	5.86 (1.68–22.40)	3.74 (1.28–10.93)	4.94 (1.35–18.08)
**Complete response vs. progressive disease**	6.94 (0.99–48.55)	41.00 (3.65–461.03)	7.77 (0.58–104.98)	43.99 (3.07–629.57)
**Partial response vs. progressive disease**	2.89 (0.75–11.19)	8.79 (1.97–39.28)	2.90 (0.65–13.00)	8.81 (1.89–41.11)
**Stable disease vs. progressive disease**	4.39 (1.63–11.81)	7.92 (2.37–26.46)	3.52 (1.22–10.14)	6.58 (1.90–22.83)

CI = confidence interval; OR = odds ratio; SMRP = soluble mesothelin-related peptides
